# Acute Pneumococcal Pleurisy

**Published:** 1905-05-27

**Authors:** 


### Hospital Clinics.

### ACUTE PNEUMOCOCCAL PLEURISY.

It is an aphorism of an experienced physician that atypical pneumonia is of worse omen than that which conforms with the characteristic clinical picture of the disease. The explanation of this fact no doubt varies in different cases, for, as was long ago pointed out by Dr. Gee, pneumonia is a general disease whose brunt falls most often on the lung, but not by any means always, and the features of any given case may be expected to vary according to the distribution of the stress of the affection among the various organs of the body. The extensive potentialities of the pneumococcus have received much fuller attention of late than they were previously believed to deserve, and pneumococcal invasions of the meninges, the pleuræ, the pericardium, the peritoneum, joints, and periosteum are now commonplaces of medical literature.

An interesting example of the kind of lesion which may underlie atypical pneumonia is offered by a case related by Dr. J L. Steven.[1] The facts are as follows:—A healthy male aged 27, after feeling poorly for two days, was seized with an acute pain in his right side. When seen on the day of onset his temperature was 102°; he complained of pain over his right lower ribs, and had a short dry cough. The next day the pain was still severe, percussion was impaired over the right lower chest, and the breath sounds in this area were tubular. During the next three days his condition deteriorated, and the signs of hydrothorax became established. On the sixth day of the illness a pint of turbid fluid was removed from his chest by siphonage, but he failed to improve; his temperature ran up to 107°, and he died the same day. The turbid fluid yielded the pneumococcus, and Dr. Steven is of opinion that the disease was not lobar pneumonia at all, but an acute pneumococcal pleurisy.

Such cases, though they appear to be very rare in adults, are by no means excessively infrequent in childhood, at which era of life generalisation of the pneumococcal, as of the tuberculous, infection, is most commonly to be met with. They have been described in such patients as "acute pneumococcal empyema" in contradistinction from the much commoner chronic pneumococcal empyemata which follow pneumonia, and occasionally appear as sequels of infectious fevers, especially measles, without any known antecedent involvement of the lungs. The characters of the fluid obtained from the chest in these cases is very characteristic: it is a thin turbid liquid of a faintly greenish hue, except in certain particularly virulent infections where its natural colour may be obscured by hæmorrhages. Under the microscope it is seen to contain a few pus cells, and vast numbers of pneumococci actively dividing. The importance of such a microscopic picture is of the highest, for it is a clear index of a virulent infection and of a minimal capacity for resistance on the part of the subject attacked. The pus from pneumococcal empyemata of the ordinary chronic type is thick and yellowish-green, and shows under the microscope an abundance of pus-cells, except in very chronic cases, where the cellular elements have degenerated into an amorphous fragmentary deposit: in addition, there is a considerable formation of fibrin (which supplies the gelatinous masses so commonly to be seen on the evacuation of these abscesses), and a remarkable scarcity of organisms. In these chronic cases it may even be impossible to demonstrate the pneumococcus by means of an ordinary film, so infrequent are they at times, and, when they are visible, they are often to be seen in chains, as opposed to the active diplococcal arrangement associated with the acute disease.

There is little doubt that pneumococcal pleurisy may be a primary affection, but, in the acute form at least, it seldom is so; at all events, in autopsies on such cases, it is common to find some coincident consolidation of the lung. Further, there are good grounds for the belief that these acute pneumococcal pleurisies are in truth but part of a general blood-infection by the organism, for it is no rarity to find in the dead-house small collections of similar thin pus in serous cavities other than the pleuræ, notably in the pericardial sac and in the peritoneum. All these considerations combine to point the moral that the recovery of thin pus full of actively dividing diplococci from the chest in an acute illness is of the worst possible significance.

[1] Glasg. Med. Jour., May 1905.

